# Association Between Lifestyle Factors and Self-Reported Cardiovascular Disease Among Canadian Adults Aged 35 Years and Older: A Study Based on Data From the Canadian Community Health Survey

**DOI:** 10.7759/cureus.104372

**Published:** 2026-02-27

**Authors:** Chinelo R Chukwudulue, Adedoyin Olawoye, Joseph E Igetei, Fatima M Adeyanju, Akinyele Oladimeji, Abimbola E Arisoyin

**Affiliations:** 1 Health, Bank Medical Centre, Ottawa, CAN; 2 Internal Medicine, Maimonides Medical Center, New York, USA; 3 General Medicine, International University of Health Sciences, Basseterre, KNA; 4 Medical School, Northern Ontario School of Medicine University, Sudbury, CAN; 5 Family Medicine, Obafemi Awolowo University, Ile-Ife, NGA; 6 Psychiatry, NYC Health + Hospitals/Harlem, New York, USA

**Keywords:** canadian community health survey, cardiovascular disease, diabetes, lifestyle factors, obesity, socioeconomic status

## Abstract

Background: Cardiovascular disease (CVD) remains a leading cause of morbidity and mortality in Canada, with lifestyle behaviors and social factors playing a central role in disease risk. Contemporary population-based evidence is essential to inform prevention strategies using nationally representative data.

Objective: The objective of the study was to examine the association between lifestyle factors and self-reported CVD among Canadian adults using the 2022 Canadian Community Health Survey (CCHS) public-use microdata file.

Methods: A cross-sectional analysis was conducted using the 2022 CCHS public-use microdata file. The study included non-institutionalized adults aged 35 years and older. Self-reported CVD, defined as heart disease and/or stroke, was the outcome of interest. Lifestyle factors included smoking status, alcohol consumption, and body mass index, while age, sex, education, household income, diabetes, and province of residence were treated as covariates. Survey weights and 1,000 bootstrap replicate weights were applied to account for the complex sampling design. Descriptive analyses and survey-weighted multivariable logistic regression were performed.

Results: The final analytic sample comprised 44,977 respondents, representing 19,524,506 Canadians. Older age, male sex, overweight or obesity, diabetes, lower household income, and abstention from alcohol were independently associated with higher odds of CVD. Female sex and individuals with higher socioeconomic status demonstrated lower adjusted odds.

Conclusions: CVD among Canadian adults is strongly associated with age, metabolic health, and socioeconomic conditions. These findings highlight the continued importance of population-level prevention strategies targeting modifiable risk factors and social inequities.

## Introduction

Cardiovascular disease (CVD) is the most prevalent cause of morbidity and mortality in the global community and a major burden to the Canadian public health [1]. Ischemic heart disease, stroke, and heart failure have a substantial share of hospitalizations, long-term disability, and healthcare spending [2,3]. The incidence of CVD is steadily increasing, notwithstanding the progress made in medical management and preventive care, primarily due to population aging, urbanization, and the persistence of modifiable risk factors in driving this disease [4]. Determinants of CVD at a population level are thus necessary in understanding prevention strategies and guiding population health [5].

Lifestyle factors play a significant role in CVD development and progression [6]. Poor eating habits, alcohol abuse, sedentary lifestyle, tobacco use, and physical inactivity have been associated with poor cardiovascular outcomes over the years [7]. On the other hand, exercise, healthy nutrition, healthy body weight, and non-smoking are well known to minimize cardiovascular risk [8,9]. This is especially significant since they are modifiable and thus the main targets of primary and secondary prevention [8]. Nevertheless, the distribution and combined influence of these lifestyle factors differ among populations because of disparities in socioeconomic status, education, cultural practices, and availability of health-promotional resources [10].

Although a large body of literature has defined the linkage between lifestyle practices and CVD, most studies have concentrated on individual risk factors or clinical cohorts instead of the populations [11]. Also, the modifications of the societal behavior, the population health interventions, and the awareness of cardiovascular risk over time require the continuous assessment of these relationships with the latest data [10]. COVID-19 and its consequences also affected lifestyle behaviors, such as physical activity, diet, and consumption of alcohol, which may have caused changes in cardiovascular risk profiles in Canadian adults [12].

Self-reported CVD is a potentially biased, but frequently used and convenient measure in large population surveys [13]. The strength of this modality includes the ability to measure the disease burden in various population groups and to study the relationship between behavioral and social determinants of health [14]. Self-reported data, when viewed with caution, can offer valuable information on a population-level regarding the trends and differences in cardiovascular health [15]. In Canada, health behavior and chronic disease surveillance are important national activities that the country depends on in monitoring the health of the population, as well as identifying the high-risk groups [16,17]. 

Considering the substantial role of CVD and the fact that most of the risk factors associated with it are modifiable, it is crucially important to consider the relationship between lifestyle habits and self-reported CVD based on the latest national data [18]. This study employs the 2022 Canadian Community Health Survey (CCHS) public-use microdata file. The CCHS is one of the most extensive sources of health-related information on Canadians, which is carried out by the Canada Statistics department [19]. It is valuable in terms of testing associations between lifestyle variables and self-reported CVD in real-life contexts, where everyday behaviors and health outcomes are tested rather than only in a clinical trial setting [20]. The objective of this study was to examine the association between lifestyle factors and self-reported CVD among Canadian adults using the 2022 CCHS public-use microdata file.

## Materials and methods

Study design and data source

This study employed a cross-sectional design using data from the 2022 CCHS public-use microdata file [21]. The CCHS is a nationally representative survey conducted by Statistics Canada that collects self-reported information on health status, chronic conditions, health behaviors, and sociodemographic characteristics of individuals living in private households across Canada. The 2022 cycle includes respondents from all provinces and territories and applies a complex, multistage sampling design to ensure national coverage. The public-use microdata file was selected because it provides population-level estimates while maintaining respondent confidentiality.

Study population

The study population included non-institutionalized Canadian adults aged 35 years and older. This age restriction was applied because the CVD variable in the 2022 CCHS public-use microdata file is defined only for respondents in this age group. Limiting the analysis accordingly ensured consistency with the survey’s target population for this outcome variable. Respondents younger than 35 years were excluded because CVD information is not collected for this age group in the public-use dataset. After applying age eligibility criteria and excluding observations with missing data on key study variables, the final analytic sample included 44,977 respondents. Survey design features were incorporated using the person-level survey weight (FWGT) to generate population-representative estimates. Based on weighted analyses, the study sample represents approximately 19,524,506 non-institutionalized Canadian adults aged 35 years and older.

Variables and measures

The primary outcome of interest was self-reported CVD, defined using the CCHS-derived indicator that captures the presence of heart disease and/or stroke. Respondents were categorized as having CVD or not based on this variable.

The main exposure variables were lifestyle factors, including smoking status, alcohol consumption, and body mass index (BMI). Smoking status was categorized as current, former, or never smoker using the traditional CCHS definition. Alcohol consumption was classified based on drinking behavior in the past 12 months as regular drinker, occasional drinker, or non-drinker. BMI was assessed using the CCHS-adjusted adult BMI classification and grouped into underweight/normal weight and overweight/obese categories.

Although physical activity was initially considered, it was excluded from the final analysis because the available physical activity indicator (active transportation in the past seven days) contained a very high proportion of missing or inapplicable responses, rendering it unsuitable for reliable population-level inference.

Covariates were selected a priori based on their established associations with CVD. These included age group (35-49 years, 50-64 years, and 65 years and older), sex, highest level of educational attainment (less than secondary school, secondary school graduate, and post-secondary education), household income (five categories ranging from less than $20,000 to $80,000 or more), province or territory of residence, and self-reported diabetes status.

Missing data

Missing data were assessed for all variables considered in the analysis. Physical activity was excluded from the study due to substantial missingness exceeding 95%, largely attributable to the survey’s skip patterns and universe restrictions. Among variables retained in the final analysis, missingness was low to moderate: educational attainment (3.23%), household income (1.32%), smoking status (2.95%), alcohol consumption (0.40%), BMI (4.79%), and diabetes status (1.35%). Age, sex, province of residence, and CVD status had no missing values. Given the relatively low proportion of missing data among included variables, a complete-case analysis approach was applied.

Statistical analysis

All analyses accounted for the complex survey design of the CCHS. Person-level survey weights were applied to generate population-representative estimates, and bootstrap replicate weights provided with the PUMF were used for variance estimation to account for the multistage sampling design.

Descriptive analyses were first conducted to summarize respondent characteristics overall and stratified by CVD status. Results were presented as weighted frequencies and weighted percentages. Group differences between respondents with and without CVD were assessed using survey-adjusted F-tests based on bootstrap replicate weights, as recommended for the analysis of CCHS public-use microdata.

Multivariable survey-weighted logistic regression was then performed to examine the association between lifestyle factors and self-reported CVD while adjusting for sociodemographic and clinical covariates. Multicollinearity among independent variables was evaluated prior to model estimation using the variance inflation factor (VIF). VIF values ranged from 1.04 to 9.49, with a mean VIF of 3.37, indicating no evidence of problematic multicollinearity. All statistical analyses were conducted using Stata version 18 (StataCorp, College Station, Texas, United States), and statistical significance was assessed using a two-sided alpha level of 0.05.

Ethical considerations

This study used publicly available, de-identified secondary data from Statistics Canada. As the CCHS public-use microdata file does not contain personal identifiers and poses no risk to respondent confidentiality, research ethics board approval was not required. The study was conducted in accordance with applicable guidelines for the ethical use of secondary survey data.

## Results

Table 1 presents the weighted baseline characteristics of Canadian adults aged 35 years and older, stratified by self-reported CVD status. The table summarizes demographic, socioeconomic, and lifestyle characteristics using population-representative estimates derived from the 2022 CCHS public-use microdata file. All estimates account for the complex survey design and bootstrap replicate weights.

**Table 1:** Baseline characteristics of non-institutionalized Canadian adults aged 35 years and older by CVD status Values are presented as weighted population counts and row percentages. Group differences between respondents with and without cardiovascular disease (CVD) were assessed using survey-adjusted F-tests based on bootstrap replicate weights; therefore, conventional chi-square statistics and p-values are not reported. CAD: Canadian dollar Table generated by the authors using Stata 18 [22].

Variable	No CVD (n = 17,826,563)	CVD (n = 1,697,943)
Gender, n (%)		
Male	8,643,845 (89%)	1,041,534 (11%)
Female	9,182,718 (93%)	656,408 (7%)
Age group (years), n (%)		
35–49	6,534,378 (98%)	110,300 (2%)
50–64	6,291,627 (94%)	386,200 (6%)
≥65	5,000,558 (81%)	1,201,442 (19%)
Education level, n (%)		
Less than secondary	878,914 (81%)	201,035 (19%)
Secondary	2,816,127 (88%)	389,216 (12%)
Post-secondary	14,131,522 (93%)	1,107,692 (7%)
Household income category (CAD), n (%)		
< 20,000	409,994 (87%)	63,695 (13%)
20,000–39,999	1,646,402 (84%)	306,142 (16%)
40,000–59,999	2,011,464 (87%)	309,003 (13%)
60,000–79,999	1,993,920 (90%)	226,806 (10%)
≥ 80,000	11,764,783 (94%)	792,297 (6%)
Smoking status, n (%)		
Current	2,082,409 (92%)	173,276 (8%)
Former	4,905,552 (88%)	691,446 (12%)
Never	10,838,602 (93%)	833,221 (7%)
Alcohol consumption in past 12 months, n (%)		
Regular	10,488,812 (92%)	856,550 (8%)
Occasional	3,199,771 (91%)	298,696 (9%)
Did not drink	4,137,980 (88%)	542,696 (12%)
Body mass index category (CCHS), n (%)		
Underweight/Normal weight	5,564,623 (93%)	446,877 (7%)
Overweight/Obese	12,261,940 (91%)	1,251,066 (9%)
Diabetes, n (%)		
No	16,267,974 (93%)	1,260,654 (7%)
Yes	1,558,588 (78%)	437,289 (22%)
Province or territory of residence, n (%)		
Newfoundland and Labrador	247,078 (88%)	33,163(12%)
Prince Edward Island	71,926 (91%)	7,523 (9%)
Nova Scotia	467,279 (89%)	55,585 (11%)
New Brunswick	370,110 (91%)	38,182 (9%)
Quebec	4,263,637 (92%)	376,939 (8%)
Ontario	6,854,936 (91%)	667,886 (9%)
Manitoba	564,979 (93%)	45,120 (7%)
Saskatchewan	460,092 (91%)	43,339 (9%)
Alberta	1,997,493 (91%)	194,255 (9%)
British Columbia	2,478,301 (91%)	233,201 (9%)
Yukon/Northwest Territories/Nunavut	50,732 (95%)	2,750 (5%)

In the weighted study population, CVD was more prevalent among male than female individuals. Among men, 1,041,534 (11%) individuals reported CVD compared with 656,408 (7%) women. Age showed a strong gradient, with CVD prevalence increasing markedly across age groups. Among adults aged 35-49 years, only 110,300 (2%) individuals reported CVD, whereas prevalence increased to 386,200 (6%) individuals among those aged 50-64 years and to 1,201,442 (19%) individuals among those aged 65 years and older.

Educational attainment was inversely associated with CVD prevalence. Individuals with less than secondary school education had the highest proportion of CVD, with 201,035 (19%) individuals reporting the condition, compared with 389,216 (12%) individuals among secondary school graduates and 1,107,692 (7%) individuals among those with post-secondary education. A similar socioeconomic pattern was observed for household income. CVD prevalence was highest among individuals with household incomes below Canadian dollar (CAD) 20,000, where 63,695 (13%) individuals reported the condition, and lowest among those with incomes of CAD 80,000 or more, among whom 792,297 (6%) individuals reported CVD.

Lifestyle-related factors also differed by CVD status. Former smokers had a higher prevalence of CVD, with 691,446 (12%) individuals affected, compared with current smokers (173,276; 8%) and never smokers (833,221; 7%). With respect to alcohol consumption, individuals who did not drink alcohol in the past 12 months had a higher prevalence of CVD (542,696; 12%) compared with regular drinkers (856,550; 8%) and occasional drinkers (298,696; 9%). CVD prevalence was slightly higher among individuals classified as overweight or obese, with 1,251,066 (9%) individuals reporting CVD, compared with 446,877 (7%) individuals among those with underweight or normal weight.

The presence of diabetes was strongly associated with CVD. Among individuals with diabetes, 437,289 (22%) reported CVD, compared with 1,260,654 (7%) individuals among those without diabetes. Geographic variation in CVD prevalence was observed across provinces and territories, with the highest proportions seen in Newfoundland and Labrador 33,163 (12%) and Nova Scotia 55,585 (11%), and the lowest in Yukon, Northwest Territories, and Nunavut 2,750 (5%).

Table 2 presents the adjusted associations between selected demographic, socioeconomic, and lifestyle factors and self-reported CVD derived from survey-weighted multivariable logistic regression analysis. Estimates reflect population-representative associations after simultaneously adjusting for all covariates included in the model and accounting for the complex survey design and bootstrap replicate weights.

**Table 2:** Adjusted odds ratios for self-reported CVD from survey-weighted multivariable logistic regression (Canadian Community Health Survey) Self-reported CVD (heart disease and/or stroke) is the binary outcome variable. Estimates were obtained from survey-weighted multivariable logistic regression using 1,000 bootstrap replicate weights. Reference categories were: male (sex), age <50 years (age group), current smoker (smoking status), regular drinker (alcohol consumption), underweight/normal weight (body mass index), no diabetes, less than secondary education (education), household income < CAD 20,000, and Newfoundland and Labrador (province). Table generated by the authors using Stata 18 [22]. CVD: cardiovascular disease; aOR: adjusted odds ratio; CI: confidence interval; CAD: Canadian dollar; NWT

Variable	aOR	95% CI	p-value
Sex			
Female	0.55	0.49–0.62	<0.001
Age group (years)			
50–64	3.24	2.19–4.79	<0.001
≥65	11.19	7.61–16.46	<0.001
Smoking status			
Former	1.14	0.93–1.40	0.211
Never	0.86	0.70–1.07	0.180
Alcohol consumption			
Occasional drinker	1.12	0.95–1.31	0.179
Did not drink in past 12 months	1.26	1.10–1.45	0.001
Body mass index			
Overweight/obese	1.24	1.08–1.42	0.002
Diabetes			
Yes	2.05	1.78–2.36	<0.001
Education			
Secondary graduate	0.93	0.77–1.12	0.449
Post-secondary	0.84	0.70–1.01	0.057
Household income (CAD)			
20,000–39,999	0.68	0.48–0.96	0.029
40,000–59,999	0.64	0.45–0.90	0.010
60,000–79,999	0.56	0.40–0.79	0.001
≥ 80,000	0.49	0.35–0.68	<0.001
Province			
Prince Edward Island	0.86	0.44–1.66	0.651
Nova Scotia	1.01	0.74–1.39	0.931
New Brunswick	0.84	0.59–1.18	0.304
Quebec	0.79	0.61–1.03	0.087
Ontario	0.99	0.76–1.29	0.957
Manitoba	0.77	0.55–1.08	0.132
Saskatchewan	0.93	0.63–1.36	0.693
Alberta	1.11	0.83–1.48	0.486
British Columbia	0.97	0.73–1.29	0.852
Yukon/Northwest Territories/Nunavut	0.64	0.43–0.96	0.032

From the findings above, it is evident that women were independently associated with substantially lower odds of CVD compared with males (adjusted OR (aOR) 0.55, 95% CI 0.49-0.62). Age demonstrated the strongest association with CVD in the model. Compared with adults younger than 50 years, individuals aged 50-64 years had more than threefold higher odds of CVD (aOR 3.24, 95%CI 2.19-4.79), while those aged 65 years and older had over elevenfold higher odds (aOR 11.19, 95%CI 7.61-16.46).

Smoking status was not significantly associated with CVD after multivariable adjustment. Former smokers had slightly higher odds compared with current smokers, though this association did not reach statistical significance (aOR 1.14, 95%CI 0.93-1.40), and never smokers had modestly lower odds (aOR 0.86, 95%CI 0.70-1.07). Alcohol consumption showed a differential pattern. Occasional drinking was not significantly associated with CVD; however, individuals who reported no alcohol consumption in the past 12 months had higher odds of CVD compared with regular drinkers (aOR 1.26, 95%CI 1.10-1.45).

BMI was independently associated withCVD, with overweight or obese individuals exhibiting higher odds compared with those of underweight or normal weight (aOR 1.24, 95%CI 1.08-1.42). Diabetes was one of the strongest correlates of CVD in the model. Individuals with diabetes had more than twice the odds of CVD compared with those without diabetes (aOR 2.05, 95%CI 1.78-2.36).

Educational attainment was inversely associated with CVD, although associations were modest. Compared with individuals with less than secondary education, those with post-secondary education showed a trend toward lower odds of CVD that approached statistical significance (aOR 0.84, 95%CI 0.70-1.01), while secondary school graduates did not differ significantly from the reference group. Household income demonstrated a clear graded association with CVD. Compared with individuals with household income below CAD 20,000, progressively lower odds of CVD were observed across increasing income categories, with the strongest protective association among those earning CAD 80,000 or more (aOR 0.49, 95%CI 0.35-0.68).

After adjustment for individual-level factors, most provinces and territories did not differ significantly from Newfoundland and Labrador with respect to CVD odds. However, residents of Yukon, Northwest Territories, and Nunavut exhibited significantly lower odds of CVD (aOR 0.64, 95%CI 0.43-0.96). No statistically significant differences were observed for the remaining provinces.

**Figure 1:**
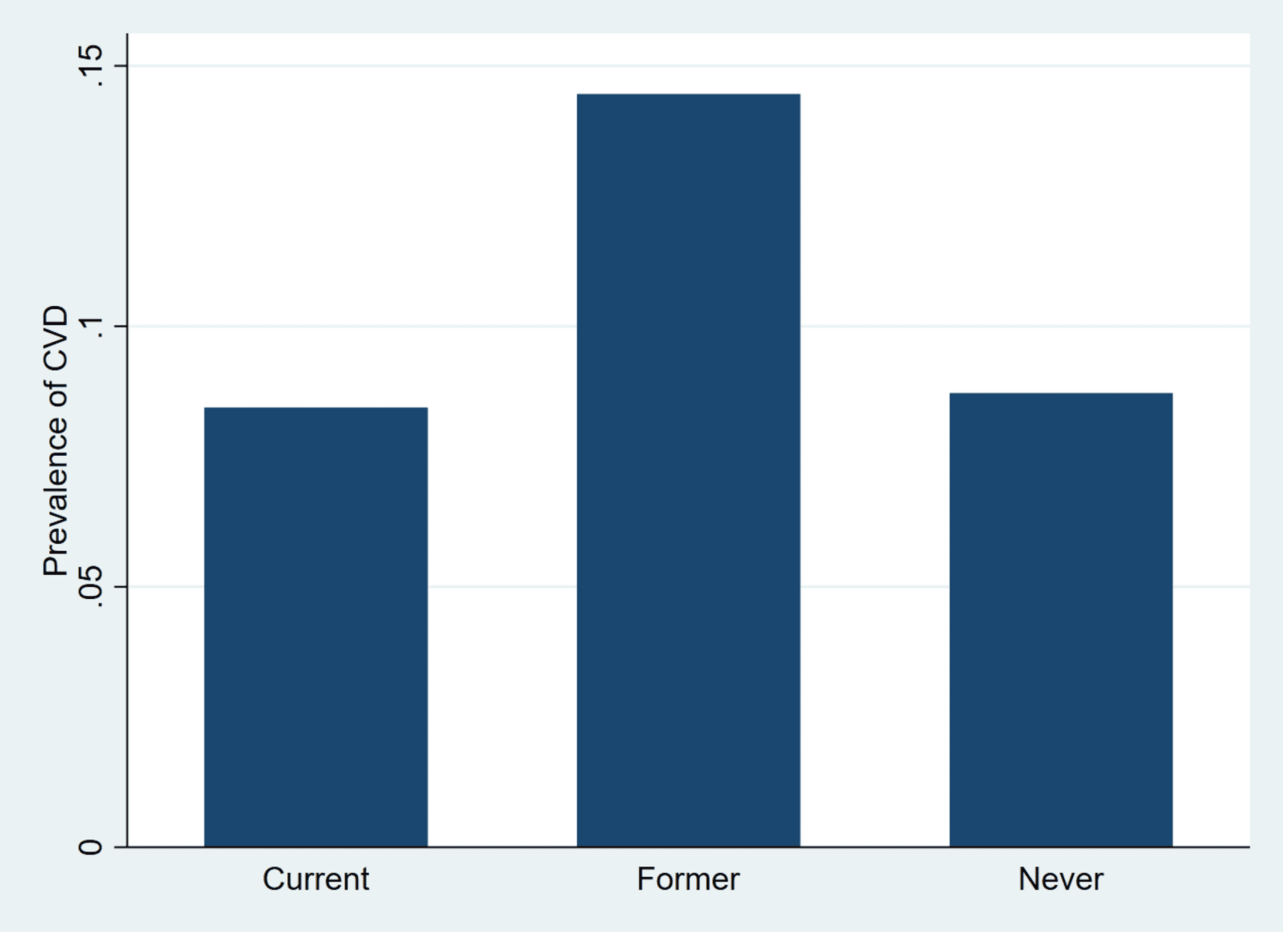
Weighted prevalence of cardiovascular disease by smoking status among Canadian adults aged ≥35 years Prevalence estimates are weighted to represent the non-institutionalized Canadian population aged 35 years and older. Smoking status was categorized as current, former, or never smoker based on self-reported responses in the Canadian Community Health Survey. All estimates account for the complex survey design and bootstrap replicate weights. CVD: cardiovascular disease Figure generated by the authors using Stata 18 [22].

Figure 1 presents the weighted prevalence of CVD according to smoking status among adults aged 35 years and older in the CCHS.

**Figure 2:**
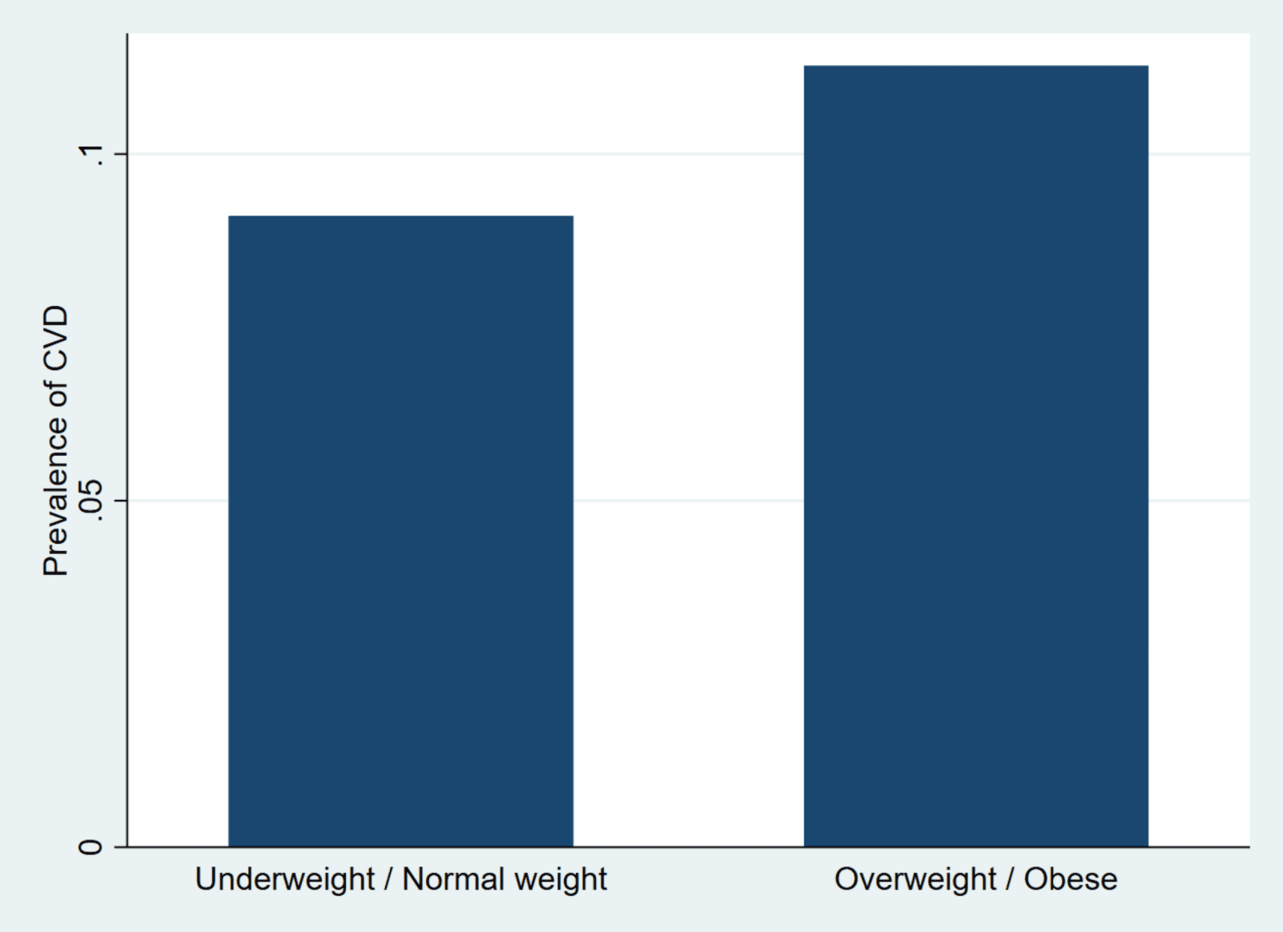
Weighted prevalence of cardiovascular disease by body mass index category among Canadian adults aged ≥35 years Prevalence estimates are weighted to represent the non-institutionalized Canadian population aged 35 years and older. Body mass index categories were derived using Canadian Community Health Survey classifications. Estimates account for the complex survey design and bootstrap replicate weights. Figure generated by the authors using Stata 18 [22].

As illustrated in Figure 1, former smokers exhibited the highest weighted prevalence of CVD compared with current smokers and those who had never smoked. The prevalence among former smokers was notably higher, while current smokers and never smokers showed similar and comparatively lower prevalence levels. This pattern likely reflects age and risk accumulation among individuals who quit smoking after prolonged exposure, as well as the higher likelihood of smoking cessation following a cardiovascular diagnosis. Consistent with the multivariable regression results, smoking status was not independently associated with CVD after adjustment for sociodemographic and clinical factors, suggesting that the observed differences in unadjusted prevalence may be partly explained by confounding factors such as age and comorbidity burden rather than current smoking behavior alone. Figure 2 visually illustrates the weighted prevalence of CVD by BMI category among adults aged 35 years and older in the CCHS.

As shown in Figure 2, the prevalence of CVD was higher among individuals classified as overweight or obese compared with those who were underweight or of normal weight. Adults in the underweight or normal weight category exhibited a lower weighted prevalence of CVD, whereas those in the overweight or obese category demonstrated a visibly greater proportion affected by CVD. This pattern is consistent with the multivariable regression findings in Table 2, which indicated that overweight or obese individuals had significantly higher adjusted odds of CVD compared with those of normal or underweight status.

## Discussion

In this study, the findings reveal that CVD risk in this population is strongly influenced by age, metabolic conditions, and socioeconomic position, with additional contributions from sex, BMI, diabetes, and alcohol consumption patterns. Collectively, these results emphasize the multifactorial nature of CVD and the continued importance of modifiable lifestyle and social determinants in population cardiovascular health.

Age was the most influential factor associated with CVD. Adults aged 65 years and older had substantially higher odds of reporting CVD compared with younger age groups. This pattern is consistent with well-established evidence linking aging to cumulative vascular injury, prolonged exposure to risk factors, and structural cardiovascular changes that increase susceptibility to ischemic heart disease, stroke, and heart failure [1-4]. Vascular aging is characterized by structural and functional changes in the blood vessels, including increased arterial stiffness and reduced elasticity. These alterations impair the ability of the vasculature to accommodate pulsatile blood flow, thereby elevating systolic blood pressure and myocardial workload, which ultimately predispose individuals to cardiovascular events [1,4]. Endothelial dysfunction, a hallmark of vascular aging, further exacerbates cardiovascular risk by diminishing nitric oxide bioavailability, promoting inflammation, and facilitating atherogenesis [2,4]. The clinical implications of these age-related vascular changes emphasize the critical importance of targeted prevention and early intervention strategies in older adults. Recognizing the progressive nature of vascular aging and endothelial impairment highlights the need for aggressive management of modifiable risk factors to slow disease progression and improve outcomes [3,4].

Sex-based differences were also observed, with women exhibiting lower odds of CVD than men after multivariable adjustment. This finding aligns with prior epidemiologic studies and may reflect biological factors such as hormonal influences, differences in fat distribution, and historical differences in risk behaviors [8,9]. Women often have lower odds of CVD than men due to a combination of biological protection and differences in fat distribution. In premenopausal women, estrogen provides cardioprotective effects by improving vascular function, lipid metabolism, and endothelial health, which delays the development of atherosclerosis and reduces CVD risk compared with men of similar age. Women also tend to store more subcutaneous fat, whereas men are more likely to accumulate visceral fat, which is strongly associated with insulin resistance, inflammation, and higher cardiovascular risk. In addition to these biological factors, cardiovascular risk profiles differ between sexes, as traditional risk factors such as hypertension, dyslipidemia, and smoking may affect men earlier or more severely, while women experience unique risk influences related to reproductive history and menopause. These differences highlight the importance of sex-specific risk assessment, screening, and prevention strategies to improve early detection and optimize cardiovascular care [8-10].

Overweight and obesity were independently associated with higher odds of CVD. Excess adiposity contributes to metabolic dysregulation, systemic inflammation, insulin resistance, and hypertension, all of which accelerate atherosclerotic processes [6,11]. Overweight and obesity (especially predominant visceral adipose tissues) contribute to CVD through several pathophysiologic mechanisms. These mechanisms include chronic inflammation, insulin resistance, hypertension, and metabolic dyslipidemia, which all contribute to the formation of atherosclerosis. Nutrient excess and excess adipose tissue activate pro-inflammatory markers, releasing interleukin (IL)-1β production and cytokine cascades [23]. This promotes oxidative stress and vascular injury, contributing to the development of atherosclerotic plaque. Obesity-related insulin resistance contributes to hyperglycemia and dyslipidemia, which are established cardiovascular risk factors associated with atherosclerosis [11,24]. Additionally, hypertension in obesity is mediated by increased sympathetic nervous system activation, renal sodium retention, and activation of the renin-angiotensin-aldosterone system, which together increase vascular resistance and cardiac workload, thereby elevating cardiovascular risk [25]. The high prevalence of overweight and obesity observed mirrors national trends and reinforces weight management as a critical component of CVD prevention in Canada [18,19].

Diabetes demonstrated a strong association with CVD, consistent with its role as a major cardiovascular risk equivalent. Chronic hyperglycemia and associated metabolic disturbances contribute to endothelial dysfunction and accelerated atherosclerosis, substantially increasing cardiovascular risk [4,6]. Alcohol consumption showed a delicate association. Individuals reporting no alcohol intake had higher odds of CVD compared with regular drinkers, likely reflecting reverse causation, whereby individuals reduce or stop alcohol consumption following a cardiovascular diagnosis [6,14]. This finding highlights the limitations of cross-sectional data in disentangling behavioral changes from disease onset.

Finally, clear socioeconomic gradients were evident, with higher education and income associated with lower CVD risk. These findings are consistent with literature linking socioeconomic advantage to healthier behaviors, improved access to care, and reduced chronic stress exposure [5,10]. Smoking status was not independently associated after adjustment, likely reflecting confounding by age and comorbidity and the challenges of temporal inference in self-reported, cross-sectional data [13,15].

Strengths and limitations

This study has several notable strengths. It utilized a large, nationally representative dataset with rigorous survey design and bootstrap replicate weights, allowing for robust population-level inference. However, this study acknowledged a number of limitations. First, the cross-sectional design prevents causal inference and limits the ability to determine temporal relationships between lifestyle behaviors and CVD. Second, CVD status was self-reported, which may introduce recall bias or underreporting. Third, physical activity was excluded from the final analysis due to extensive missing data exceeding 95%, limiting the ability to assess its contribution despite its established relevance to cardiovascular health [6,12]. Finally, residual confounding by unmeasured factors such as diet quality, medication use, and family history cannot be ruled out.

Future studies should prioritize longitudinal designs to better clarify causal pathways between lifestyle behaviors and CVD. Improved measurement of physical activity and dietary patterns in national surveys would enhance the assessment of modifiable risk factors.

## Conclusions

This nationally representative study demonstrates that self-reported CVD among Canadian adults aged 35 years and older is strongly associated with advancing age, diabetes, overweight or obesity, and lower socioeconomic status. Lower odds of CVD were observed among females and individuals with higher education and income levels, highlighting persistent social gradients in cardiovascular health. Alcohol abstinence was associated with higher CVD prevalence, likely reflecting behavioral changes following diagnosis rather than a protective effect of alcohol. Smoking status was not independently associated after adjustment, underscoring the influence of age and comorbidity in cross-sectional analyses. These findings reinforce the multifactorial nature of CVD and emphasize the importance of integrated prevention strategies that address metabolic health and social determinants. Continued surveillance using population-based data is essential to guide equitable CVD prevention and policy planning in Canada.
